# Association between renal function trajectories and risk of cardiovascular disease: a prospective cohort study

**DOI:** 10.1080/07853890.2024.2427907

**Published:** 2024-12-01

**Authors:** Xuehong Xu, Rulin Ma, Xianghui Zhang, Heng Guo, Mulatibieke Keerman, Xinping Wang, Yu Li, Remina Maimaitijiang, Jia He, Shuxia Guo

**Affiliations:** a Department of Public Health, Shihezi University School of Medicine, Shihezi, China; b Department of National Health Commission Key Laboratory of Prevention and Treatment of Central Asia High Incidence Diseases, The First Affiliated Hospital of Shihezi University Medical College, Shihezi, China

**Keywords:** Renal function trajectory, glomerular filtration rate, cardiovascular disease, cohort study

## Abstract

**Introduction:**

It is unclear whether changing trajectories of renal function will increase the risk prediction information of cardiovascular disease (CVD). This study aimed to evaluate the trajectory patterns of estimated glomerular filtration rate (eGFR) and the association between eGFR trajectories and CVD risk.

**Methods:**

A total of 4742 participants were included in the cohort from the 51st Regiment of Xinjiang Production and Construction Corps. The study endpoint was the occurrence of CVD events. eGFR trajectories were identified using a linear mixed-effects model in four distinct patterns. Multivariate Cox proportional hazards models analysed the correlations between eGFR trajectories and CVD.

**Results:**

During a median follow-up period of 5.7 years, a total of 559 (11.8%) CVD, 404 (8.5%) myocardial infarction (MI), 244 (5.2%) ischemic stroke (IS), and 62 (1.3%) heart failure (HF) incidents occurred. After multivariable adjustment, gradual decline trajectory increased the risk of CVD (*HR* 1.42, 95% *CI* 1.16–1.74), MI (*HR* 1.41, 95% *CI* 1.11–1.79), and IS (*HR* 1.41, 95% *CI* 1.04–1.92); gradual increase trajectory reduced the risk of CVD (*HR* 0.40, 95% *CI* 0.25–0.64) and MI (*HR* 0.49, 95% *CI* 0.29–0.81). Consistent results were obtained in sensitivity and subgroup analyses.

**Conclusions:**

Decline and increase of renal function were related to the risk of CVD, MI, and IS in the rural areas of Xinjiang. Monitoring eGFR changing trajectory is of great significance in improving the risk of CVD.

## Introduction

The prevalence of cardiovascular disease (CVD) continues to increase globally and remains a heavy disease burden [1]. With the proposal of the concept of ‘Cardiorenal Syndrome,’ the close relationship between renal function and CVD has drawn extensive attention [2]. The data show that approximately 35% of the patients with acute kidney injury are commonly complicated with CVD, and approximately half of the patients with chronic kidney disease (CKD) die of CVD [3,4].

Estimated glomerular filtration rate (eGFR) is a good estimation of GFR for evaluating renal function, renal damage, and CKD level [5]. Several studies have evaluated the effectiveness of eGFR in predicting adverse clinical outcomes. Meta-analyses showed a linear relationship between the single measurements of eGFR and CVD mortality in patients with eGFR < 75 mL/min/1.73 m^2^ [6]. Several reports have indicated that persons with reduced renal function are at a higher risk for CVD events such as myocardial infarction (MI), stroke (IS), heart failure (HF), and peripheral arterial disease, both in the general population and high-risk groups [7–9]. The results of these studies suggest that eGFR is an emerging risk factor for CVD and a predictor of CVD risk [10].

Currently, Clinical Practice Guidelines suggest evaluating the risk of adverse outcomes by monitoring the changing trend in eGFR over time [9,11]. Therefore, some studies used the eGFR slope and percentage change in eGFR to identify the association between eGFR change and end-stage renal disease [12], CVD [13] and all-cause mortality [14]. However, this indicator is calculated using only two measured values and does not consider the non-linear changes and time effects of eGFR. The influence of time-dependent changes is largely ignored. Based on this, the concept of eGFR trajectories was proposed [15]. eGFR trajectories refer to the dynamically changing tendencies of eGFR, which are fitted by multiple-measurement eGFR data. eGFR trajectories can provide more information on renal condition and are the best method to estimate renal function [16]. Several population-based studies have evaluated the association between changes of renal function over time and adverse outcomes [17,18]. However, only a few studies took CVD as the outcome variable, and these results were uncertain [19,20]. Second, the declining trajectory of eGFR is associated with CVD, especially in patients with CKD [7], hypertension [21] and diabetes [14]. However, it has not yet been confirmed in the general population with relatively normal renal function [22].

Xinjiang is a multiethnic area located in Northwest China. The Uighurs are the largest ethnic group in Xinjiang and have a unique cultural background and genetic characteristics. Some studies have found a high rate of exposure to CVD risk factors such as obesity among the Uighurs; the prevalence of CVD is also higher among them [23]. Can we apply eGFR indicators, especially eGFR trajectories, to predict the CVD risk? Therefore, the aim of this study is to (1) identify the trajectory patterns of eGFR in the population in rural areas of Xinjiang and (2) evaluate the association between the eGFR trajectories and CVD risk. Identifying abnormal eGFR trajectories as early as possible is important for the high-risk population to take effective and timely interventions to reduce the future burden of CVD.

## Materials and methods

### Study design and population

This cohort study was conducted in Kashgar, Xinjiang. Using typical sampling, the 51st Regiment of the Farm, the only one regiment with Uyghurs as its main residents, was selected as the survey site. The baseline survey was conducted from 2016, and four follow-ups from 2019-2022, until the end of August 2022 (follow-up rate: 92.6%). Among the 6576 Uighur adults recruited, we excluded frequent floating populations, pregnant women, individuals who were unable to participate in the survey process and participants with incomplete basic information. We further excluded participants with chronic kidney disease, kidney failure and kidney transplant at baseline; participants with a history of cardiovascular disease. Therefore, a total of 5585 individuals were included in the baseline survey. Participants who lost follow-up and those who measured serum creatinine less than 3 times were excluded. Finally, a total of 4742 individuals were included in the trajectory analysis (Supplementary Figure 1). The Institutional Ethics Review Board of the First Affiliated Hospital of Shihezi University School of Medicine approved this study (IERB no.: 2016-121-01), and all participants provided written informed consent.

### Data collection and definitions

The research data were collected through a standardized questionnaire, physical examination, and laboratory testing. Through face-to-face interviews, the questionnaire was used to collect information on age, gender, smoking, drinking, complications, and family history. The survey was performed by investigators who had received professional training. Trained professionals used standardized methods to measure height, weight, waist circumference (WC), and hip circumference (HC). Body mass index (BMI) was calculated according to the formula: BMI = weight (kg)/height (m^2^). After the participants sat for at least 5 min, an automatic blood pressure meter (HBP-9020, Omron (China) Co., Ltd.) measured three blood pressures and recorded the average value of these measurements.

After the participants had fasted for at least 6 h, venous blood samples were collected into a vacuum tube in the morning. An automatic biochemical analyser (Olympus AU 2700; Olympus Diagnostics, Hamburg, Germany) was used to measure fasting blood glucose (FBG), serum creatinine (Scr), total cholesterol (TC), triglycerides (TG), high-density lipoprotein cholesterol (HDL-C) and low-density lipoprotein cholesterol (LDL-C). All tests were performed by experienced doctors at the central laboratory of the First Affiliated Hospital of Shihezi University Medical College. eGFR was calculated using the Chronic Kidney Disease Epidemiology Collaboration (CKD-EPI) and Modification of Diet in Renal Disease (MDRD) formulas [24].

In this study, hypertension was defined as systolic blood pressure (SBP) ≥ 140 mmHg or diastolic blood pressure (DBP) ≥ 90 mmHg, and diabetes was defined as FBG ≥ 7.0 mmol/L, with previous diagnosis of hypertension/diabetes and use of blood pressure/glucose control drugs [25,26]. Overweight and obesity was defined as BMI ≥ 24.0 kg/m^2^ [27]. Dyslipidaemia was defined as TC ≥ 6.2, TG ≥ 2.3 or HDL-*C* ≤ 1.0 [28]. The definition of a family history of hypertension was that at least one parent or sibling has a history of hypertension, and the same criteria were used for family history of diabetes, coronary heart disease, and stroke. According to the World Health Organization recommendations, smoking was defined as smoking for more than 6 months [29]. Drinking was defined as drinking at least twice a month [30].

### Assessments of eGFR trajectory pattern

In this study, different trajectory patterns of eGFR were considered as exposure. We used the CKD-EPI and MDRD formulas every year to calculate eGFR [24]. The individual trajectories of the participants were fitted according to the mixed-effect model and classified into four distinct patterns: eGFR high-level stable progress trajectory (T0), eGFR gradual decline trajectory (T1), eGFR low-level slow increase trajectory (T2), and eGFR gradual increase trajectory (T3).

### Study outcomes and follow-up

The outcome events in this study were the new cardiovascular events during the follow-up period from 2017 to 2022. The International Classification of Diseases codes (ICD-10) [31] were used for outcome classification, namely myocardial infarction (MI, I21-I22), ischemic stroke (IS, I60-I64, I69), heart failure (HF, I50), arrhythmia, and peripheral arterial disease. The CVD events were recorded using inpatient and social security records. Participants were followed until the CVD event of concern occurred, individual was lost to follow-up, or the study ended.

### Statistical analysis

We prospectively obtained the eGFR data; participants had varied numbers of eGFR tests, but were guaranteed ≥ 3 times. A linear mixed-effects model was applied to fit individual trajectories to repeatedly measured eGFR values using the R package *lme4*. We obtained Empirical Bayes Estimates of the fixed and random effects for each individual. Using the corresponding estimated fixed and random effects as coefficients, the trajectories of individuals can be fitted using quadratic and cubic polynomials. Thus, the eGFR trajectory for each participant was modelled as a linear quadratic and cubic function from the baseline to the end of the follow-up period. Finally, a probabilistic clustering procedure was applied to cluster the individual trajectories, which was implemented using the R package *mclust*. The number of clustered trajectories was defined based on Bayesian Information Criterion. The results showed four trajectory patterns for eGFR.

Baseline characteristics were summarized as means and standard deviations for continuous variables and numbers and percentages for categorical variables. One-way analysis of variance or Kruskal–Wallis test was used for comparison of continuous variables and chi-square test for comparison of categorical variables. Multiple comparisons of subgroups were made using post-hoc analysis with Bonferroni correction.

The cumulative risk of CVD was assessed using Kaplan–Meier analysis for each trajectory group and compared using log-rank tests. Cox proportional risk models were used to compare the relationship between eGFR trajectories and the risk of developing each type of cardiovascular outcome, and to estimate the calculated *HR* and its corresponding 95% *CI*. Multivariate regression analysis was performed using the forward stepwise method for variables that were significant in univariate Cox regression analysis. Variables were retained in the final model if the main predictor variable (eGFR trajectory) was changed by at least 5%. The final complete model determined using the stepwise method was adjusted for age, sex, smoking, SBP, WC, diabetes, and family history of coronary heart disease and diabetes.

We performed a series of sensitivity analyses to test the robustness of the results. First, the MDRD formula was applied to recalculate the eGFR and perform a sensitivity analysis to describe the robustness of the eGFR trajectory model. Next, those with hypertension, diabetes, overweight/obesity, and dyslipidemia were excluded to test the robustness of the primary outcome. Finally, we performed a subgroup analysis. Consistency of association of eGFR trajectory patterns with risk of CVD development was assessed for men and women and in subgroup analyses stratified by age < 45 years and ≥ 45 years.

All analyses were performed using SPSS version 26 (SPSS Inc., Chicago, IL, USA) and R version 4.2.2(R Foundation for Statistical Computing, Vienna, Austria). Statistical tests were two-sided, and statistical significance was set at *p* < 0.05.

## Results

### The trajectory of eGFR

**Figure 1.**
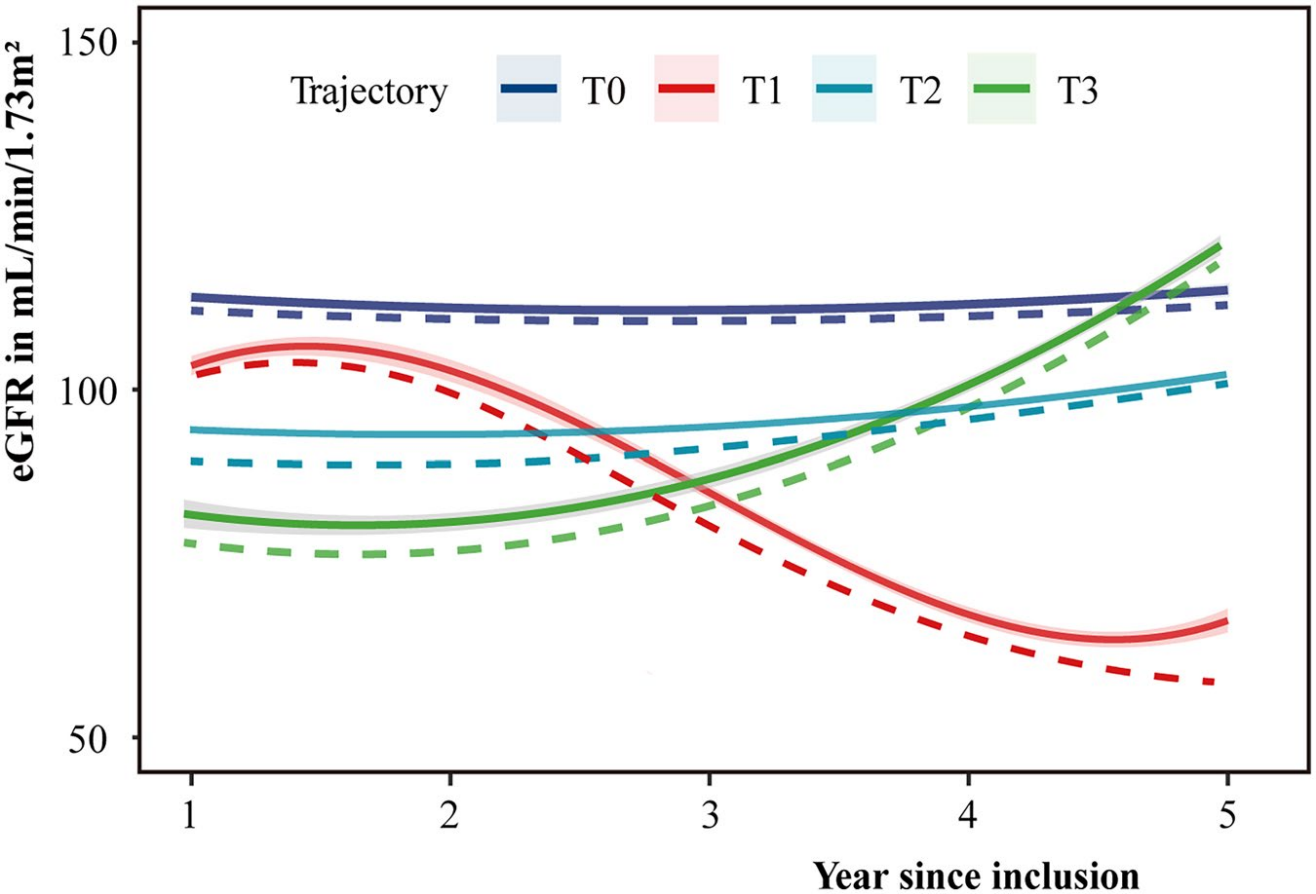
Changing trajectory patterns of eGFR. The solid lines represented the eGFR value calculated using the CKD-EPI formula, and the dashed lines represented the eGFR value calculated using the MDRD formula. eGFR estimated glomerular filtration rate.

This study determined four sets of different eGFR trajectory patterns (Figure 1): T0 (*n* = 2187, 46.1%) was characterized by a baseline eGFR of 113 mL/min/1.73 m^2^, and an estimated eGFR of 112 mL/min/1.73 m^2^ after a median follow-up of 5.71 years, which is defined as a stable high-level progress trajectory. T0 represented no changes in renal function and progression of renal disease. T1 (*n* = 1012, 21.3%) was characterized by a baseline eGFR of 106 mL/min/1.73 m^2^. There was no significant decline in the early stage, and then it continued to decline > −3 mL/min/1.73 m^2^/year. T2 (*n* = 1065, 22.5%) was characterized by a baseline eGFR of 94 mL/min/1.73 m^2^, stable progress in the first year of follow-up, and slowly increase in the following three years. T3 (*n* = 478, 10.1%) showed a baseline eGFR of 77 mL/min/1.73 m^2^, which has not increased significantly in early time, and continued to increase > 3 mL/min/1.73 m^2^/year.

### Baseline characteristics

Table 1 shows the baseline characteristics of the 4,742 participants based on the different eGFR trajectory patterns. Among them, females comprised 58.67% of the cohort. The mean age was 39.98 (13.30) years, mean SBP was 128.07 (20.09) mmHg, and 70.52% of the participants were classified as overweight or obese (BMI ≥ 24 kg/m^2^). In total, 24.72% had hypertension, 6.05% had diabetes, and 22.20% had a family history of chronic disease. A total of 559 (11.79%) CVD, 404 (8.52%) MI, 244 (5.15%) IS, and 62 (1.31%) HF events occurred during the median follow-up of 5.7 years.

**Table 1.** Baseline characteristics of the study population of different eGFR trajectory patterns.

Variables	Total	eGFR trajectory patterns			
		T0	T1	T2	T3
N (%)	4742	2187 (46.12)	1012 (21.34)	1065 (22.46)	478 (10.08)
Age, years	39.98 (13.30)	37.60 (11.67)	45.66 (14.77)^a^	40.14 (13.51)^a^^,b^	38.53 (12.65)^b^
Female	2782 (58.67)	1114 (50.94)	665 (65.71)^a^	657 (61.69)^a^	346 (72.38)^a^^,c^
Smoking	576 (12.15)	272 (12.44)	133 (13.14)	136 (12.77)	35 (7.32)^a^^,b,c^
Drinking	164 (3.46)	74 (3.38)	30 (2.96)	53 (4.98)	7 (1.46)^c^
BMI, kg/m^2^	27.00 (5.03)	26.55 (4.84)	27.59 (5.08)^a^	27.00 (5.03)	27.71 (5.26)^a^
HC, cm	99.95 (9.99)	99.83 (10.01)	100.38 (10.03)	99.55 (9.69)	100.42 (10.44)
WC, cm	92.75 (12.84)	92.62 (12.81)	94.07 (12.90)^a^	91.86 (12.54)^b^	92.56 (13.37)
SBP, mmHg	128.07 (20.09)	126.24 (18.02)	133.39 (23.10)^a^	128.71 (20.25)^a^^,b^	123.77 (19.59)^b^^,c^
DBP, mmHg	76.56 (11.50)	75.66 (10.99)	79.14 (12.64)^a^	76.58 (11.03)^b^	75.16 (11.46)^b^
Laboratory results					
TC, mmol/L	4.68 (1.05)	4.62 (1.10)	4.79 (0.97)^a^	4.74 (1.07)^a^	4.64 (0.92)^b^
TG, mmol/L	1.69 (1.20)	1.58 (1.16)	1.81 (1.27)^a^	1.79 (1.34)^a^	1.67 (0.86)
LDL-C, mmol/L	2.69 (0.95)	2.78 (0.93)	2.67 (1.02)^a^	2.60 (1.00)^a^	2.52 (0.71)^a^^,b^
HDL-C, mmol/L	1.40 (0.53)	1.40 (0.57)	1.41 (0.55)^a^	1.40 (0.47)^a^	1.40 (0.34)^a^^,b^
FBG, mmol/L	5.04 (1.97)	4.91 (1.84)	5.25 (2.19)^a^	5.21 (1.97)^a^	4.91 (1.14)^b^
eGFR, mL/min/1.73m^2^	103 (24)	113 (13)	106 (33)^a^	94 (21)^a^^,b^	77 (18)^a^^,b,c^
History					
Hypertension	1172 (24.72)	459 (20.99)	346 (34.19)^a^	274 (25.73)^a^^,b^	93 (19.46)^b^^,c^
Diabetes	287 (6.05)	104 (4.76)	80 (7.91)^a^	84 (7.89)^a^	19 (3.97)^b^^,c^
Family history					
Hypertension	705 (14.87)	360 (16.46)	152 (15.02)	164 (15.40)	29 (6.07)^a^^,b,c^
Diabetes	253 (5.34)	116 (5.30)	62 (6.13)	65 (6.10)	10 (2.09)^a^^,b,c^
Coronary heart disease	389 (8.20)	184 (8.41)	88 (8.70)	103 (9.67)	14 (2.93)^a^^,b,c^
Ischemic stroke	186 (3.92)	85 (3.89)	47 (4.64)	48 (4.51)	6 (1.26)^a^^,b,c^
Outcomes					
CVD	559 (11.79)	202 (9.23)	226 (22.33)	111 (10.42)	20 (4.18)
MI	404 (8.52)	148 (6.77)	158 (15.61)	81 (7.61)	17 (3.56)
IS	244 (5.51)	80 (3.66)	112 (11.07)	39 (3.66)	13 (2.72)
HF	62 (1.31)	16 (0.73)	27 (2.67)	13 (1.22)	6 (1.26)

*Notes:* Data were shown as means (standard deviations), or numbers (proportions %). BMI: body mass index; HC: hip circumference; WC: waist circumference; SBP: systolic blood pressure; DBP: diastolic blood pressure; TC: total cholesterol; TG: triglycerides; LDL-C: low-density lipoprotein cholesterol; HDL-C: high-density lipoprotein cholesterol; FBG: fasting blood glucose; eGFR: estimated glomerular filtration rate; MI: myocardial infarction; IS: ischemic stroke; HF: heart failure; T0: eGFR high-level stable progress trajectory; T1: eGFR gradual decline trajectory; T2: eGFR low-level slow increase trajectory; T3: eGFR gradual increase trajectory.

^a^Compared to T0 group, the difference was statistically significant.

^b^Compared to T1 group, the difference was statistically significant.

^c^Compared to T2 group, the difference was statistically significant.

*p < 0.05.

After *post hoc* testing of all pairs of subgroups, participants in the eGFR gradual decline trajectory (T1) were older, had a higher proportion of smokers, and had higher DBP and triglyceride levels than participants in the other subgroups. Age, SBP, total cholesterol, baseline eGFR, and family history of disease were lower in the eGFR gradual increase trajectory (T3) than in the other subgroups.

### Association of eGFR trajectory pattern and cardiovascular disease

**Figure 2.**
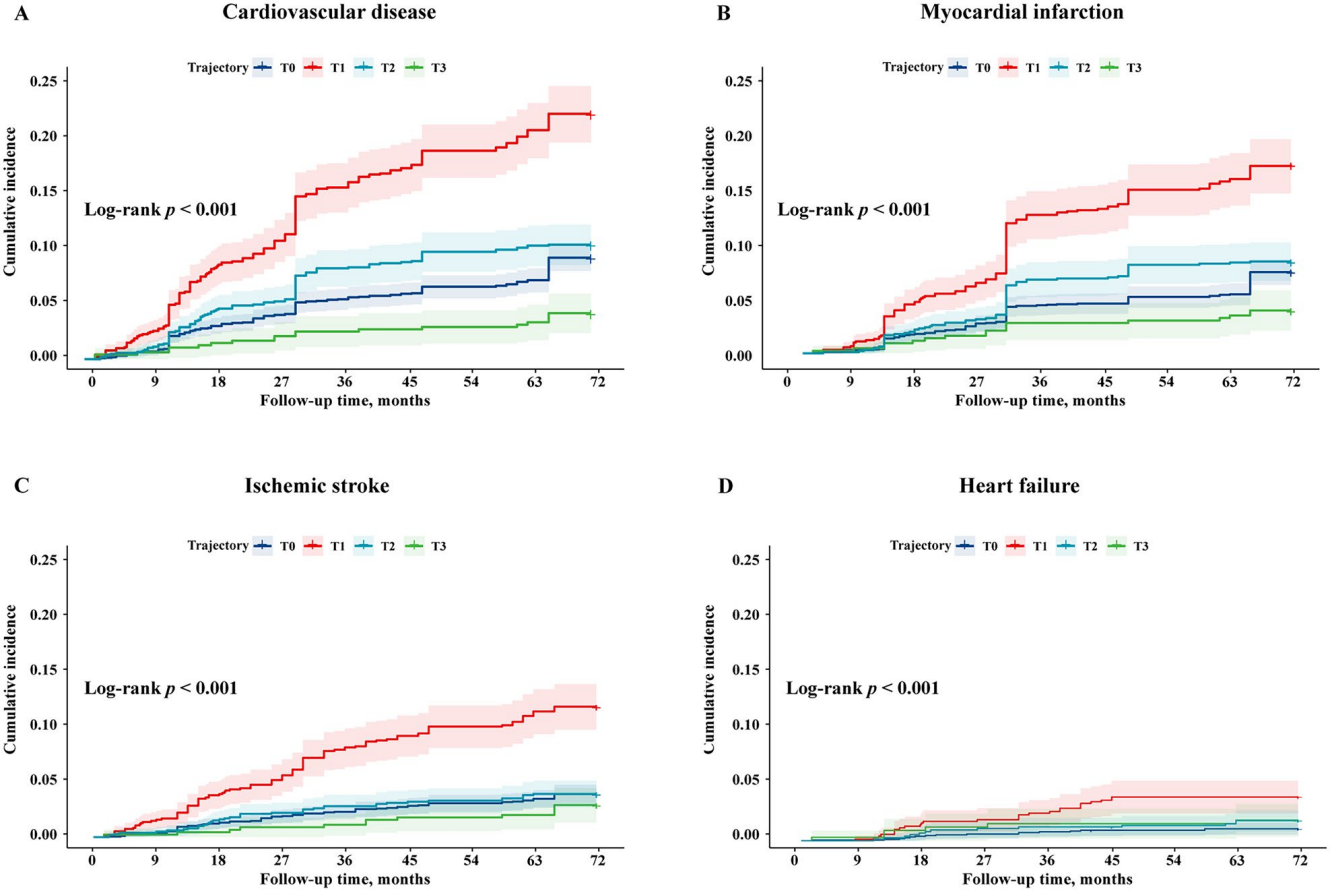
Kaplan–Meier curves of cumulative incidence of cardiovascular disease (a), myocardial infarction (B), ischemic stroke (C), and heart failure (D) according to eGFR trajectory patterns.

The Kaplan–Meier curves in Figure 2 show that, with T0 as the reference group, the cumulative incidence of total CVD events was higher in the T1 group (χ^2^=108.10; *p* < 0.001; *HR*: 2.62, 95% *CI* 2.17–3.17) and lower in the T3 group (χ^2^=12.86; *p* < 0.001; *HR*: 0.44, 95% *CI* 0.28–0.70). Similar trends were observed for the HF and IS events. Of these, the cumulative incidence of MI (χ^2^=65.14; *p* < 0.001; *HR*: 2.43, 95% *CI* 1.94–3.04) and IS (χ^2^=68.51; *p* < 0.001; *HR*: 3.15, 95% *CI* 2.36–4.19) was higher in the T1 group. In contrast, MI was lower in the T3 group (χ^2^=6.80; *p* < 0.001; *HR*: 0.52, 95% *CI* 0.32–0.86).

Table 2 shows the corrected incidence of MI, IS, HF, and CVD according to the eGFR trajectory classification. Compared with the participants with stable high-level trajectory, the gradual decline trajectory group increased the risk of CVD (*HR*: 2.62, 95% *CI* 2.17–3.17), MI (*HR*: 2.43, 95% *CI* 1.94–3.04), IS (*HR*: 3.15, 95% *CI* 2.36–4.19), and HF (*HR*: 3.68, 95% *CI* 1.98–6.83). In contrast, the gradual increase trajectory grou*p* reduced the risks of CVD (*HR*: 0.44, 95% *CI* 0.28–0.70) and MI (*HR*: 0.52, 95% *CI* 0.32–0.86). After adjusting for covariates, the gradual decline trajectory group (highest incidence observed) was still associated with a high risk of developing CVD (*HR*: 1.42, 95% *CI* 1.16–1.74), MI (*HR*: 1.41, 95% *CI* 1.11–1.79), and IS (*HR*: 1.41, 95% *CI* 1.04–1.92). In contrast, the gradual increase trajectory group had a reduced risk of CVD (*HR*: 0.40, 95%*CI* 0.25–0.64) and MI (*HR*: 0.49, 95% *CI* 0.29–0.81).

**Table 2.** HRs And 95%CIs for risk of cardiovascular disease events according to eGFR trajectory patterns.

		T1		T2		T3	
	T0	*HR* (95%*CI*)	*P*-values	*HR* (95%*CI*)	*P*-values	*HR* (95%*CI*)	*P*-values
CVD							
Case/1000 person-year	15.3	37.2		17.3		7.0	
Model 1	Ref	2.62 (2.17–3.17)	<0.001	1.15 (0.91–1.45)	0.24	0.44 (0.28–0.70)	0.001
Model 2	Ref	1.47 (1.21–1.80)	<0.001	0.89 (0.71–1.13)	0.35	0.37 (0.24–0.59)	<0.001
Model 3	Ref	1.42 (1.16–1.74)	0.001	0.85 (0.67–1.08)	0.18	0.40 (0.25–0.64)	<0.001
MI							
Case/1000 person-year	11.3	26.0		12.7		6.0	
Model 1	Ref	2.43 (1.94–3.04)	<0.001	1.14 (0.87–1.49)	0.35	0.52 (0.32–0.86)	0.01
Model 2	Ref	1.44 (1.13–1.82)	0.003	0.90 (0.69–1.19)	0.47	0.45 (0.28–0.75)	0.002
Model 3	Ref	1.41 (1.11–1.79)	0.005	0.88 (0.67–1.16)	0.36	0.49 (0.29–0.81)	0.005
IS							
Case/1000 person-year	6.2	18.5		6.2		4.5	
Model 1	Ref	3.15 (2.36–4.19)	<0.001	1.01 (0.68–1.47)	0.99	0.74 (0.41–1.33)	0.31
Model 2	Ref	1.48 (1.09–2.01)	0.01	0.71 (0.48–1.05)	0.09	0.61 (0.34–1.09)	0.09
Model 3	Ref	1.41 (1.04–1.92)	0.03	0.66 (0.45–0.98)	0.04	0.67 (0.37–1.20)	0.17
HF							
Case/1000 person-year	1.2	4.5		2.0		2.2	
Model 1	Ref	3.68 (1.98–6.83)	<0.001	1.67 (0.80–3.48)	0.17	1.72 (0.67–4.40)	0.27
Model 2	Ref	1.54 (0.79–3.01)	0.21	1.13 (0.54–2.39)	0.74	1.46 (0.57–3.75)	0.43
Model 3	Ref	1.47 (0.75–2.87)	0.27	1.06 (0.50–2.25)	0.89	1.58 (0.62–4.07)	0.34

*Note:* Data were shown as *HR*, 95%*CI*, and *P*-value. Model 1: unadjusted; Model 2: adjusted age and sex; Model 3: further adjusted smoking, SBP, WC, Diabetes, family history of Diabetes and coronary heart disease.

CVD: cardiovascular disease; MI: myocardial infarction; IS: ischemic stroke; HF: heart failure; T0: eGFR high-level stable progress trajectory; T1: eGFR gradual decline trajectory; T2: eGFR low-level slow increase trajectory; T3: eGFR gradual increase trajectory; Ref: reference group; HR: hazard ratio; CI: confidence interval.

In addition to trajectory, the univariate Cox models showed that the risk of CVD outcomes were significantly associated with female, higher age, BMI, HC, WC, SBP, DBP, TC, TG, LDL-C, HDL-C, FBG, having hypertension, having diabetes, and having a family history of chronic disease(Supplementary Table 1). In the multivariate analyses, the risk of CVD outcomes increased with age by approximately 6% per year, and women had a higher risk of disease than men. Smoking and WC also increased the risk of CVD, except for IS. Other factors that were significantly associated with stroke include SBP and DBP. Hypertension was associated with HF events. Diabetes and family history of coronary heart disease were related to MI and CVD. The remaining variables were not associated with an increased CVD risk (Supplementary Table 2).

### Sensitivity and subgroup analysis

**Figure 3.**
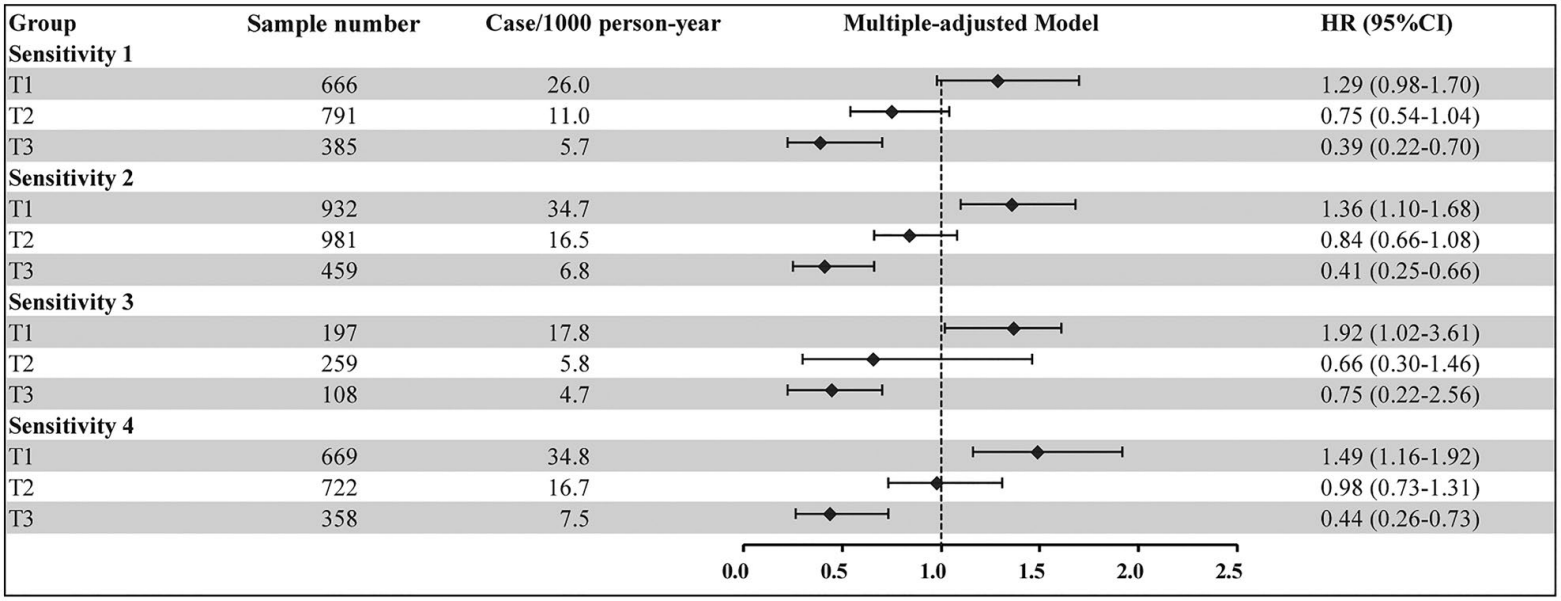
Sensitivity analysis of the relationship between eGFR trajectory patterns and cardiovascular disease. *Notes:* Sensitivity 1: excluded participants with hypertension (n = 1172); Sensitivity 2: excluded participants with diabetes (n = 287); Sensitivity 3: excluded participants with overweight and obese (n = 3645); Sensitivity 4: excluded participants with dyslipidemia (n = 1467). Model adjusted age, sex, smoking, SBP, WC, Diabetes, family history of Diabetes and coronary heart disease. HR: hazard ratio; CI: confidence interval.

**Figure 4.**
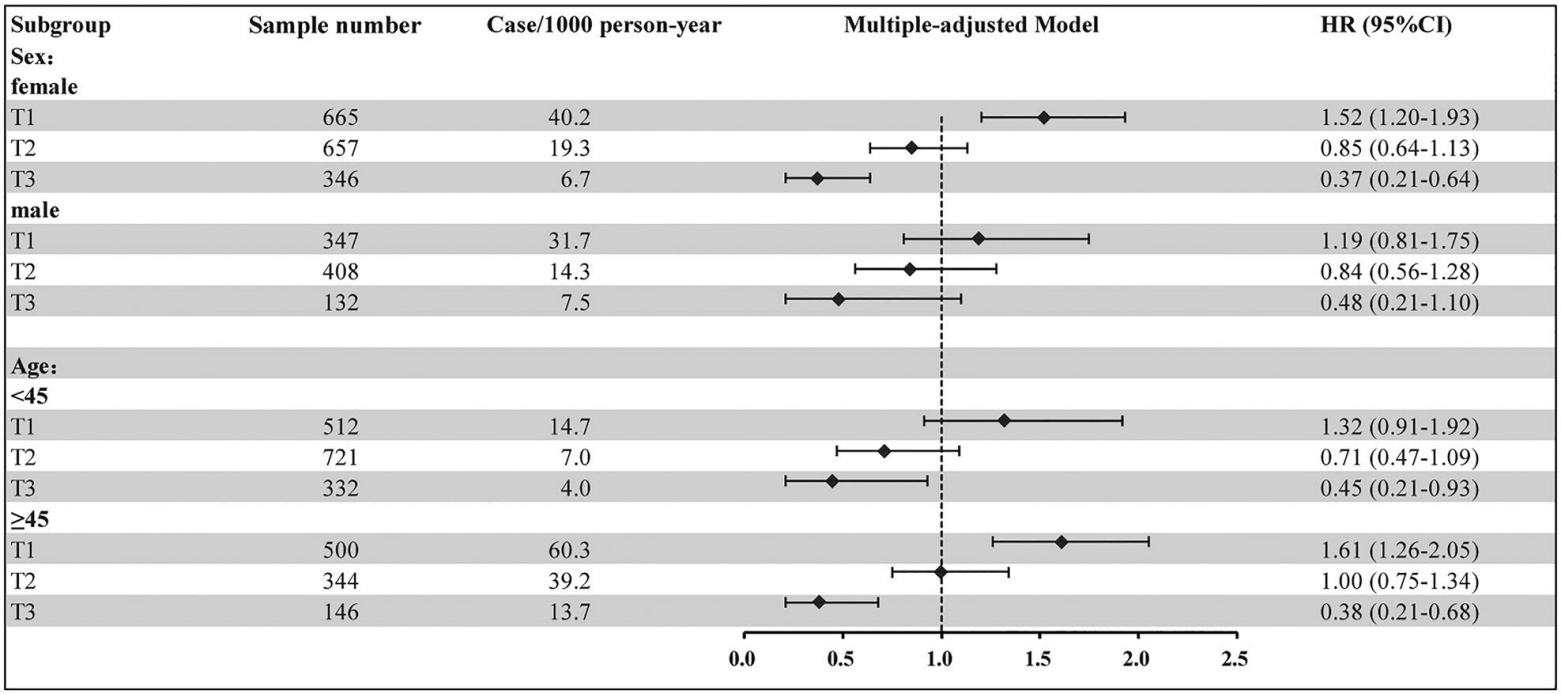
Subgroup analysis of the relationship between eGFR trajectory patterns and cardiovascular disease. *Notes:* Model adjusted age, sex, smoking, SBP, WC, Diabetes, family history of Diabetes and coronary heart disease. HR: hazard ratio; CI: confidence interval.

To test the stability of trajectory fitting, the MDRD formula was used to calculate eGFR as an alternative method for plotting the eGFR trajectory. Similar trajectories were also obtained (Figure 1). Simultaneously, to examine the robustness of our primary outcome, we performed a series of sensitivity analyses. We have excluded participants in the following status: Participants with hypertension, diabetes, overweight and obesity, and dyslipidemia. The results were similar to the main analysis (Figure 3). We also performed subgroup analysis. Similar results were obtained from the analysis of the male and female subgroups. In stratified analysis by age < 45 and ≥ 45 years, the correlation between eGFR trajectories and CVD was consistent (Figure 4). The results of sensitivity and subgroup analyses for other specific types of CVD events were shown in Supplementary Figures 2 and 3.

## Discussion

In this study, we evaluated the independent correlation of four groups of renal function trajectory patterns with CVD and three specific CVD events (MI, IS, and HF). The results showed that the gradual decline trajectory of renal function detected by eGFR was associated with a higher risk of MI, IS, and CVD events, and the gradual rise trajectory of renal function was associated with a lower risk of MI and CVD events. After multivariate adjustment and subgroup analysis, consistent results were obtained.

The study found that the cumulative incidence of CVD among the Uighur population in the rural areas of Xinjiang was 11.8%, similar to the results of European countries (12.1%) [32], but higher than that of the Chinese Han population (4.3–7.6%) [33,34]. This may be related to the unique diet, lifestyle, natural environment, and genetic characteristics of this population. First, our study population had the characteristics of hypertension [35], hyperlipidaemia, obesity [36], metabolic syndrome [37] risk factor gathering and a high incidence. Second, Uighurs have the lowest rate of ethnic intermarriage in China. Their cultural beliefs and living customs were less affected by mainstream Han culture, their population flow was low, and they had unique ethnic genes [38]. This may lead to a higher incidence of CVD. For renal function assessment, the MDRD formula was estimated to be lower than the CKD-EPI formula; however, the overall trend of the trajectory was the same. This is consistent with studies reporting that the MDRD formula underestimates GFR levels in normal populations [39,40].

In the general population, based on a single eGFR measurement, the relationship between baseline renal function and risk of end-stage renal disease, cardiovascular disease, and all-cause mortality has been well confirmed [41,42]. However, there is no consensus on whether changes in the long-term renal function trajectory are associated with CVD events. Data have suggested that, among the general population, the decline in renal function is not related to the carotid artery intima-media thickness [43], stroke [44], or arteriosclerosis [45]. However, it is worth mentioning that these studies did not monitor the changes in the dynamic trajectory of renal function. This study evaluated the association between different eGFR trajectory patterns and the risk of CVD events. Consistent with our assumptions, this study found that the trajectory of eGFR decline is associated with an increased risk of cardiovascular events. Compared with the eGFR stable trajectory, participants in the eGFR declining trajectory had a 2.62-fold (95% CI: 2.17–3.17), 2.43-fold (95% CI: 1.94–3.04) and 3.15-fold (95% CI: 2.36–4.19) increased risk of CVD, MI, and IS, respectively. Even if factors such as population statistics, laboratory indicators, and complications related to the results are adjusted, a correlation still exists. These results emphasize the importance of the eGFR decline trajectory as a risk factor for CVD and the potential value of risk prediction. The current results are in agreement with those of other studies. A cohort study by Guo et al. [46] of 37,691 adults aged 45 years or older found that participants with a rapidly declining eGFR had a higher risk of CVD than those with a stable eGFR. A community-based cohort study by Turin et al. [8] found that participants with the greatest decline (≤-5mL/min/1.73 m^2^/year) had a more than 2-fold (95% CI: 2.28–2.89) increased risk of CHF compared to those with stable eGFR. The risks of acute MI and IS increased by 31% and 29%, respectively. The following mechanisms may explain the association between reduced renal function and increased adverse cardiovascular events: the reduction in eGFR may exacerbate cardiovascular risk factors, such as blood pressure and lipid levels [47]. We can see that the blood pressure and lipid index of the eGFR decline trajectory group were higher than other trajectory groups. Other possible factors that were not measured in our cohort included RAAS activation, endothelial dysfunction, inflammatory factor release, and oxidative stress [48]. In addition, these risk factors and the progression of CVD have accelerated the progression of CKD. It is unclear whether deteriorating renal function is a specific factor contributing to poor outcomes or simply a marker of advanced heart and renal dysfunction.

This study has a unique aspect. In addition to the decline in renal function, we noticed a 60% (*HR*: 0.40, 95% *CI* 0.25–0.64) and 51% (*HR*: 0.49, 95% *CI* 0.29–0.81) lower risk of CVD and MI, respectively, in participants with a gradual increase trajectory (T3) compared to those with stable eGFR (T0). However, the current evidence is still uncertain regarding the relationship between eGFR increase and CVD risk. This finding was consistent with the results reported by Shepherd et al. [20]. This cohort study of 9,500 participants aged 35-75 years, who received atorvastatin, found a 28% reduction in CVD events in patients with stable renal function (*HR*: 0.72; 95% *CI* 0.60–0.87) and a 64% reduction in patients with improved renal function compared to patients with worsening renal function. Some cohort studies found that an increase in eGFR was not associated with CVD risk. Turin et al. [19] found that eGFR rising (> 5 mL/min/1.73 m^2^) was only related to stroke risk (*HR*: 1.23, 95% *CI* 1.05-1.44). Possible explanations for the difference in results are as follows: First, the standards used to define the increase in eGFR and the period in which the change in eGFR is based introduce heterogeneity. Second, there were differences in the design between the different cohorts. Studies that included non-healthy people with more CKD, diabetes, or basic diseases were more likely to conclude that an increase in the eGFR trajectory increased the risk of disease. In contrast, this study included a healthier population that was relatively younger and had fewer comorbidities. The difference in the populations also contributed to the difference in the results. The eGFR rising trajectory in these studies occurred when eGFR was already at a low level, and the rising trajectory in this study still fluctuated within the normal range of 80-120 mL/min/1.73 m^2^. Therefore, stabilizing renal function within the normal range may be beneficial to health. In addition, the serum creatinine value used to calculate eGFR is influenced by factors such as muscle mass, age, sex, diet, and drugs (e.g. atorvastatin), in addition to renal function. The rising trajectory of renal function included more young and male participants than the other trajectories. Owing to the high metabolic rate of young and strong individuals, the glomerular filtering ability is relatively high.

This cohort study evaluated the trajectory of renal function changes during the long-term follow-up period, which comprehensively describes the eGFR trajectory pattern of the general population. The strengths of this study include the continuous measurement of eGFR and correction for multiple important risk factors. In addition, we used a more robust linear mixed-effects model than the ordinary linear regression model to estimate the intercept and slope of the eGFR curve, which minimized residual confounding. The eGFR trajectory considers all available eGFR measurement values that individuals can obtain over time. Therefore, this model can be fitted to explain the non-linear eGFR trajectory and evaluate the changes in eGFR over time among participants with different measurement intervals.

Our study has some limitations. Firstly, we lacked the information on drugs known to be associated with the development of CVD. Although we corrected for multiple confounders, we could not exclude residual confounders such as drug treatment. Through sensitivity analysis, we minimized this confounding factor as much as possible by excluding participants with a history of hypertension and diabetes. The CVD risk did not change qualitatively. Secondly, only Uighurs were included in this study. Further research is required to test the validity of these findings in other populations. Lastly, we used eGFR instead of directly measured GFR to assess renal function, which may lead to misclassification of the true changes in renal function. However, the measured GFR is not practical for typical clinical applications or large epidemiological studies.

## Conclusion

This study observed the decline and increase in renal function were independently related to the risk of MI, IS, and CVD. The eGFR trajectory proved to be a valuable predictor of cardiovascular events. Our results suggested that monitoring the changing trajectory of renal function over time will help to identify individuals at a higher risk of adverse cardiovascular events and allow for accurate risk stratification.

## Supplementary Material

Supplemental Material

## Data Availability

The datasets used and/or analysed during the current study are available from the corresponding author on reasonable request.
