# Supplementary material for: Association between renal function trajectories and risk of cardiovascular disease: a prospective cohort study
Source: Ann Med. 2024 Dec 1;56(1):2427907. doi: 10.1080/07853890.2024.2427907 (PMC12002098; doi:10.1080/07853890.2024.2427907)
Supplement: Supplemental Material [file IANN_A_2427907_SM2499.zip › suppl_data/captions and notes.docx]

**Association between renal function trajectories and risk of cardiovascular disease: a prospective cohort study**

**Figure S1.** Flow chart of study participant inclusion and exclusion criteria.

**Figure S2.** Sensitivity analysis of the relationship between eGFR trajectory patterns and myocardial infarction (A), ischemic stroke (B), and heart failure (C).

*Notes:* Sensitivity 1: excluded participants with hypertension (n=1172); Sensitivity 2: excluded participants with diabetes (n=287); Sensitivity 3: excluded participants with overweight and obese (n=3645); Sensitivity 4: excluded participants with dyslipidemia (n=1467). Model adjusted age, sex, smoking, SBP, WC, Diabetes, family history of Diabetes and coronary heart disease. HR: hazard ratio; CI: confidence interval.

**Figure S3.** Subgroup analysis of the relationship between eGFR trajectory patterns and myocardial infarction (A), ischemic stroke (B), and heart failure (C).

*Notes:* Model adjusted age, sex, smoking, SBP, WC, Diabetes, family history of Diabetes and coronary heart disease. HR: hazard ratio; CI: confidence interval.

**Table S1.** Univariate associations of eGFR trajectory patterns with cardiovascular disease outcomes.

*Notes:* Data were shown as HR, 95% CI, and *P*-value. BMI: body mass index; HC: hip circumference; WC: waist circumference; SBP: systolic blood pressure; DBP: diastolic blood pressure; TC: total cholesterol; TG: triglycerides; LDL-C: low-density lipoprotein cholesterol; HDL-C: high-density lipoprotein cholesterol; FBG: fasting blood glucose; eGFR: estimated glomerular filtration rate; MI: myocardial infarction; IS: ischemic stroke; HF: heart failure; CVD: cardiovascular disease; T0: eGFR high-level stable progress trajectory; T1: eGFR gradual decline trajectory; T2: eGFR low-level slow increase trajectory; T3: eGFR gradual increase trajectory.

**Table S2.** Multivariate associations of eGFR trajectory patterns with cardiovascular disease outcomes.

*Notes:* Data were shown as HR, 95% CI, and *P*-value. BMI body mass index, HC hip circumference, WC waist circumference, DBP diastolic blood pressure, SBP systolic blood pressure, TC total cholesterol, TG triglycerides, LDL-C low-density lipoprotein cholesterol, HDL-C high-density lipoprotein cholesterol, FBG fasting blood glucose, eGFR estimated glomerular filtration rate, MI: myocardial infarction; IS: ischemic stroke; HF: heart failure; CVD: cardiovascular disease; T0 eGFR high-level stable progress trajectory, T1 eGFR gradual decline trajectory, T2 eGFR low-level slow increase trajectory, T3 eGFR gradual increase trajectory
