# Supplementary material for: Association between renal function trajectories and risk of cardiovascular disease: a prospective cohort study
Source: Ann Med. 2024 Dec 1;56(1):2427907. doi: 10.1080/07853890.2024.2427907 (PMC12002098; doi:10.1080/07853890.2024.2427907)
Supplement: Supplemental Material [file IANN_A_2427907_SM2499.zip › suppl_data/Table S1.docx]

| **Table S1.** Univariate associations of eGFR trajectory patterns with cardiovascular disease outcomes | | | | | | | |
| --- | --- | --- | --- | --- | --- | --- | --- |
|  | **MI (n=404)** | **IS (n=244)** | **HF (n=62)** | | **CVD (n=559)** | | |
| **Variables** | **HR (95%CI)** | **HR (95%CI)** | **HR (95%CI)** | **β** | | **HR (95%CI)** | ***P*-value** |
| Age, years | 1.06 (1.05-1.06) | 1.07 (1.06-1.08) | 1.09 (1.07-1.11) | 0.06 | | 1.06 (1.06-1.07) | ＜0.001 |
| Female | 1.35 (1.10-1.66) | 1.70 (1.29-2.24) | 0.91 (0.55-1.51) | 0.42 | | 1.52 (1.27-1.82) | ＜0.001 |
| Smoking | 1.30 (0.99-1.71) | 0.64 (0.40-1.01) | 1.75 (0.93-3.29) | 0.13 | | 1.14 (0.89-1.45) | 0.28 |
| Drinking | 0.99 (0.58-1.69) | 0.47 (0.01-1.00) | 0.05 (0.00-1.89) | -0.36 | | 0.70 (0.41-1.18) | 0.19 |
| BMI, kg/m^2^ | 1.07 (1.05-1.09) | 1.08 (1.05-1.10) | 1.11 (1.07-1.16) | 0.07 | | 1.07 (1.06-1.09) | ＜0.001 |
| HC, cm | 1.03 (1.03-1.04) | 1.03 (1.02-1.04) | 1.04 (1.02-1.06) | 0.03 | | 1.03 (1.02-1.04) | ＜0.001 |
| WC, cm | 1.04 (1.04-1.05) | 1.04 (1.03-1.05) | 1.06 (1.05-1.08) | 0.04 | | 1.04 (1.03-1.05) | ＜0.001 |
| SBP, mmHg | 1.02 (1.02-1.03) | 1.03 (1.03-1.04) | 1.03 (1.02-1.04) | 0.03 | | 1.03 (1.02-1.03) | ＜0.001 |
| DBP, mmHg | 1.03 (1.02-1.04) | 1.05 (1.04-1.05) | 1.03 (1.02-1.05) | 0.04 | | 1.04 (1.03-1.04) | ＜0.001 |
| **Laboratory results** |  |  |  |  | |  |  |
| TC, mmol/L | 1.12 (1.03-1.22) | 1.25 (1.14-1.37) | 1.22 (1.01-1.48) | 0.16 | | 1.18 (1.10-1.26) | ＜0.001 |
| TG, mmol/L | 1.06 (0.99-1.13) | 1.11 (1.03-1.19) | 1.08 (0.92-1.26) | 0.09 | | 1.09 (1.04-1.16) | 0.001 |
| LDL-C, mmol/L | 1.21 (1.12-1.31) | 1.29 (1.19-1.40) | 1.28 (1.09-1.52) | 0.22 | | 1.25 (1.17-1.33) | ＜0.001 |
| HDL-C, mmol/L | 0.73 (0.58-0.91) | 0.83 (0.63-1.08) | 0.79 (0.45-1.37) | -0.21 | | 0.81 (0.68-0.97) | 0.03 |
| FBG, mmol/L | 1.06 (1.03-1.09) | 1.06 (1.02-1.09) | 1.07 (1.01-1.14) | 0.05 | | 1.05 (1.03-1.08) | ＜0.001 |
| eGFR, mL/min/1.73m^2^ | 1.00 (0.99-1.01) | 0.99 (0.98-0.99) | 0.99 (0.98-1.00) | -0.01 | | 0.99 (0.99-1.01) | 0.50 |
| **Comorbidities** |  |  |  |  | |  |  |
| Hypertension | 2.65 (2.18-3.29) | 3.95 (3.07-5.09) | 4.55 (2.74-7.55) | 1.08 | | 2.95 (2.50-3.49) | ＜0.001 |
| Diabetes | 2.12 (1.57-2.88) | 2.17 (1.47-3.19) | 1.99 (0.91-4.38) | 0.70 | | 2.01 (1.54-2.62) | ＜0.001 |
| **eGFR trajectories** |  |  |  |  | |  |  |
| T0 | 1.0 | 1.0 | 1.0 | 1.0 | | 1.0 | 1.0 |
| T1 | 2.43 (1.94-3.04) | 3.15 (2.36-4.19) | 3.68 (1.98-6.83) | 0.96 | | 2.62 (2.17-3.17) | ＜0.001 |
| T2 | 1.14 (0.87-1.49) | 1.00 (0.68-1.47) | 1.67 (0.80-3.48) | -0.81 | | 1.15 (0.91-1.45) | ＜0.001 |
| T3 | 0.52 (0.32-0.86) | 0.74 (0.41-1.33) | 1.72 (0.67-4.40) | 0.14 | | 0.44 (0.28-0.70) | 0.24 |
| **Family history** |  |  |  |  | |  |  |
| Hypertension | 1.19 (0.92-1.54) | 1.24 (0.89-1.72) | 0.97 (0.48-1.97) | 0.27 | | 1.31 (1.06-1.62) | 0.01 |
| Diabetes | 1.57 (1.09-2.25) | 1.53 (0.96-2.44) | 1.23 (0.45-3.39) | 0.41 | | 1.50 (1.10-2.06) | 0.01 |
| Coronary heart disease | 1.76 (1.31-2.34) | 1.42 (0.95-2.11) | 1.43 (0.65-3.14) | 0.49 | | 1.63 (1.26-2.10) | ＜0.001 |
| Ischemic stroke | 1.46 (0.95-2.24) | 1.76 (1.06-2.92) | 1.71 (0.62-4.70) | 0.45 | | 1.56 (1.09-2.23) | 0.01 |
| *Notes:* Data were shown as HR, 95% CI, and *P*-value. BMI: body mass index; HC: hip circumference; WC: waist circumference; SBP: systolic blood pressure; DBP: diastolic blood pressure; TC: total cholesterol; TG: triglycerides; LDL-C: low-density lipoprotein cholesterol; HDL-C: high-density lipoprotein cholesterol; FBG: fasting blood glucose; eGFR: estimated glomerular filtration rate; MI: myocardial infarction; IS: ischemic stroke; HF: heart failure; CVD: cardiovascular disease; T0: eGFR high-level stable progress trajectory; T1: eGFR gradual decline trajectory; T2: eGFR low-level slow increase trajectory; T3: eGFR gradual increase trajectory. | | | | | | | |
