# Supplementary material for: Association between renal function trajectories and risk of cardiovascular disease: a prospective cohort study
Source: Ann Med. 2024 Dec 1;56(1):2427907. doi: 10.1080/07853890.2024.2427907 (PMC12002098; doi:10.1080/07853890.2024.2427907)
Supplement: Supplemental Material [file IANN_A_2427907_SM2499.zip › suppl_data/Table S2.docx]

| **Table S2.** Multivariate associations of eGFR trajectory patterns with cardiovascular disease outcomes. | | | | | | | |
| --- | --- | --- | --- | --- | --- | --- | --- |
|  | **MI (n=404)** | **IS (n=244)** | **HF (n=62)** | | **CVD (n=559)** | | |
| **Variables** | **HR (95%CI)** | **HR (95%CI)** | **HR (95%CI)** | **β** | | **HR (95%CI)** | ***P*-value** |
| Age, years | 1.05 (1.04-1.06) | 1.07 (1.05-1.08) | 1.08 (1.05-1.11) | 0.05 | | 1.05 (1.05-1.06) | ＜0.001 |
| Female | 1.92 (1.48-2.48) | 1.97 (1.44-2.71) | 1.26 (0.67-2.36) | 0.79 | | 2.21 (1.77-2.75) | ＜0.001 |
| Smoking | 2.34 (1.65-3.32) | 1.42 (0.86-2.37) | 3.12 (1.45-6.66) | 0.89 | | 2.43 (1.79-3.30) | ＜0.001 |
| Drinking | 1.06 (0.59-1.90) | 0.00 (0.00-4.78) | 0.00 (0.00-3.96) | -0.26 | | 0.77 (0.44-1.37) | 0.38 |
| BMI, kg/m^2^ | 0.99 (0.97-1.02) | 1.02 (0.98-1.06) | 1.03 (0.96-1.12) | 0.001 | | 1.00 (0.98-1.03) | 0.93 |
| HC, cm | 1.00 (0.98-1.02) | 0.99 (0.97-1.01) | 0.97 (0.93-1.01) | -0.004 | | 1.00 (0.98-1.01) | 0.55 |
| WC, cm | 1.03 (1.01-1.04) | 1.01 (0.99-1.03) | 1.05 (1.02-1.09) | 0.02 | | 1.02 (1.01-1.03) | 0.001 |
| SBP, mmHg | 0.99 (0.98-1.00) | 1.01 (1.00-1.02) | 0.99 (0.97-1.01) | 0.01 | | 1.01 (0.99-1.01) | 0.13 |
| DBP, mmHg | 1.01 (0.99-1.02) | 1.01 (1.00-1.03) | 1.00 (0.97-1.03) | 0.01 | | 1.01 (0.99-1.02) | 0.20 |
| **Laboratory results** |  |  |  |  | |  |  |
| TC, mmol/L | 0.93 (0.83-1.05) | 1.02 (0.88-1.18) | 1.00 (0.74-1.34) | -0.04 | | 0.96 (0.87-1.06) | 0.38 |
| TG, mmol/L | 0.97 (0.89-1.06) | 1.02 (0.94-1.11) | 0.92 (0.72-1.18) | 0.02 | | 1.02 (0.96-1.09) | 0.47 |
| LDL-C, mmol/L | 1.04 (0.93-1.15) | 1.09 (0.96-1.23) | 1.11 (0.86-1.43) | 0.06 | | 1.06 (0.98-1.16) | ＜0.17 |
| HDL-C, mmol/L | 0.88 (0.72-1.08) | 0.97 (0.76-1.23) | 0.95 (0.62-1.46) | -0.21 | | 0.98 (0.83-1.15) | 0.80 |
| FBG, mmol/L | 0.98 (0.92-1.05) | 0.95 (0.86-1.05) | 1.03 (0.88-1.20) | -0.04 | | 0.97 (0.91-1.03) | 0.29 |
| eGFR, mL/min/1.73m^2^ | 1.00 (0.99-1.01) | 1.00 (0.99-1.01) | 1.00 (0.99-1.01) | 0.002 | | 1.00 (1.00-1.01) | 0.07 |
| **History** |  |  |  |  | |  |  |
| Hypertension | 1.27 (0.92-1.75) | 0.91 (0.61-1.36) | 2.30 (1.03-5.13) | -0.01 | | 0.99 (0.76-1.31) | 0.97 |
| Diabetes | 1.72 (1.05-2.82) | 1.64 (0.86-3.14) | 0.89 (0.26-3.07) | 0.51 | | 1.66 (1.07-2.59) | 0.02 |
| **eGFR trajectories** |  |  |  |  | |  |  |
| T0 | 1.0 | 1.0 | 1.0 | 1.0 | | 1.0 | 1.0 |
| T1 | 1.45 (1.14-1.84) | 1.38 (1.01-1.88) | 1.48 (0.75-2.93) | 0.36 | | 1.43 (1.17-1.75) | 0.001 |
| T2 | 0.91 (0.69-1.20) | 0.67 (0.46-0.99) | 1.07 (0.49-6.66) | -0.12 | | 0.88 (0.70-1.12) | 0.31 |
| T3 | 0.50 (0.30-0.82) | 0.69 (0.38-1.25) | 1.51 (0.57-3.99) | -0.85 | | 0.43 (0.27-0.68) | ＜0.001 |
| **Family history** |  |  |  |  | |  |  |
| Hypertension | 1.13 (0.84-1.51) | 1.16 (0.80-1.69) | 1.15 (0.51-2.61) | 0.20 | | 1.22 (0.95-1.55) | 0.11 |
| Diabetes | 1.37 (0.93-2.01) | 1.66 (0.99-2.78) | 1.17 (0.39-3.51) | 0.33 | | 1.39 (0.99-1.95) | 0.05 |
| Coronary heart disease | 1.70 (1.20-2.41) | 1.21 (0.74-1.99) | 1.39 (0.49-3.98) | 0.36 | | 1.43 (1.06-1.96) | 0.02 |
| Ischemic stroke | 1.46 (0.95-2.24) | 1.29 (0.70-2.40) | 1.42 (0.39-5.11) | -0.04 | | 0.96 (0.63-1.46) | 0.84 |
| Data were shown as HR, 95% CI, and *P*-value.  BMI body mass index, HC hip circumference, WC waist circumference, DBP diastolic blood pressure, SBP systolic blood pressure, TC total cholesterol, TG triglycerides, LDL-C low-density lipoprotein cholesterol, HDL-C high-density lipoprotein cholesterol, FBG fasting blood glucose, eGFR estimated glomerular filtration rate, MI: myocardial infarction; IS: ischemic stroke; HF: heart failure; CVD: cardiovascular disease; T0 eGFR high-level stable progress trajectory, T1 eGFR gradual decline trajectory, T2 eGFR low-level slow increase trajectory, T3 eGFR gradual increase trajectory | | | | | | | |
