# Supplementary figures and images for: Association between renal function trajectories and risk of cardiovascular disease: a prospective cohort study
Source: Ann Med. 2024 Dec 1;56(1):2427907. doi: 10.1080/07853890.2024.2427907 (PMC12002098; doi:10.1080/07853890.2024.2427907)

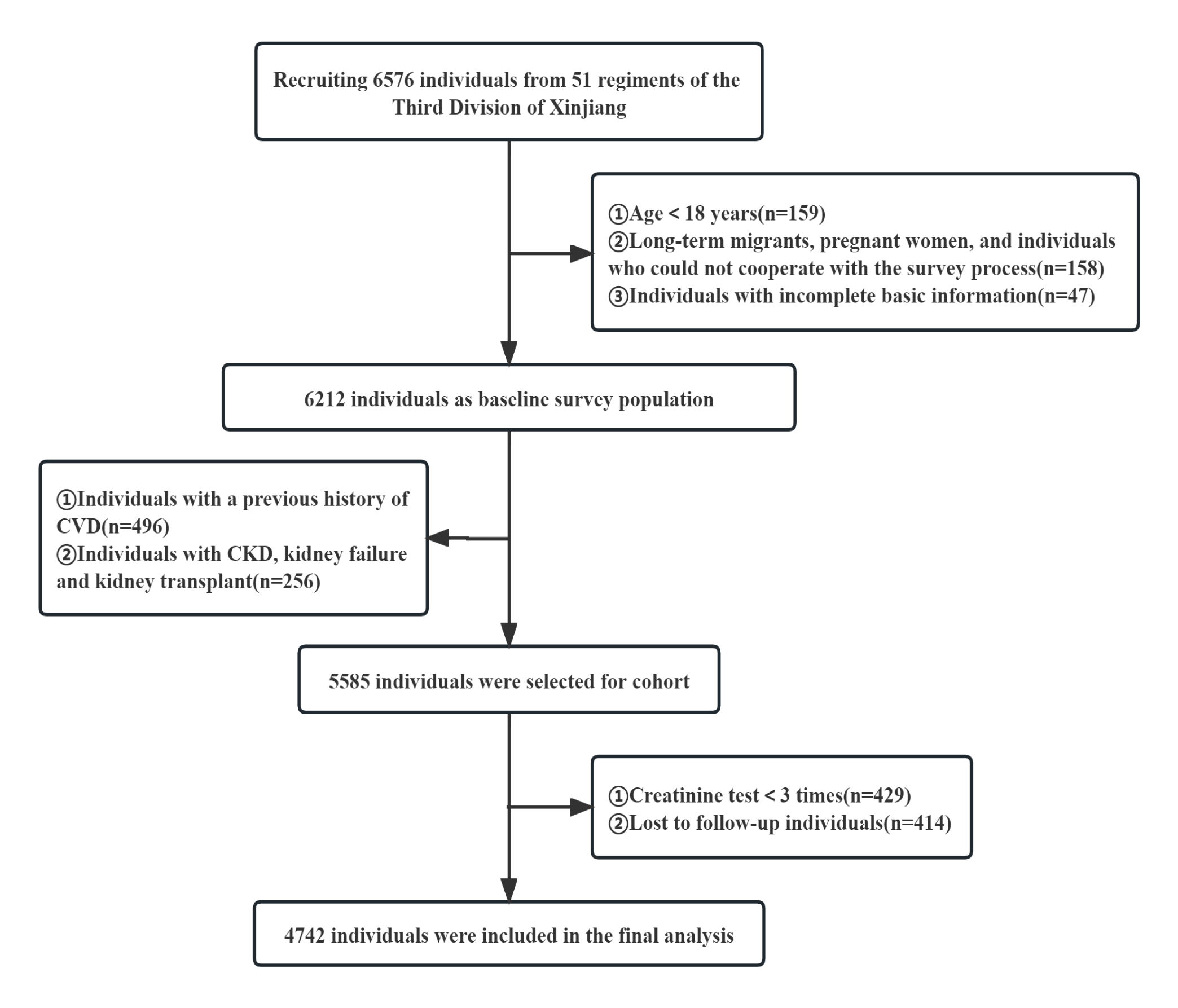

Supplement: Supplemental Material [file IANN_A_2427907_SM2499.zip › suppl_data/Figure S1.tif]

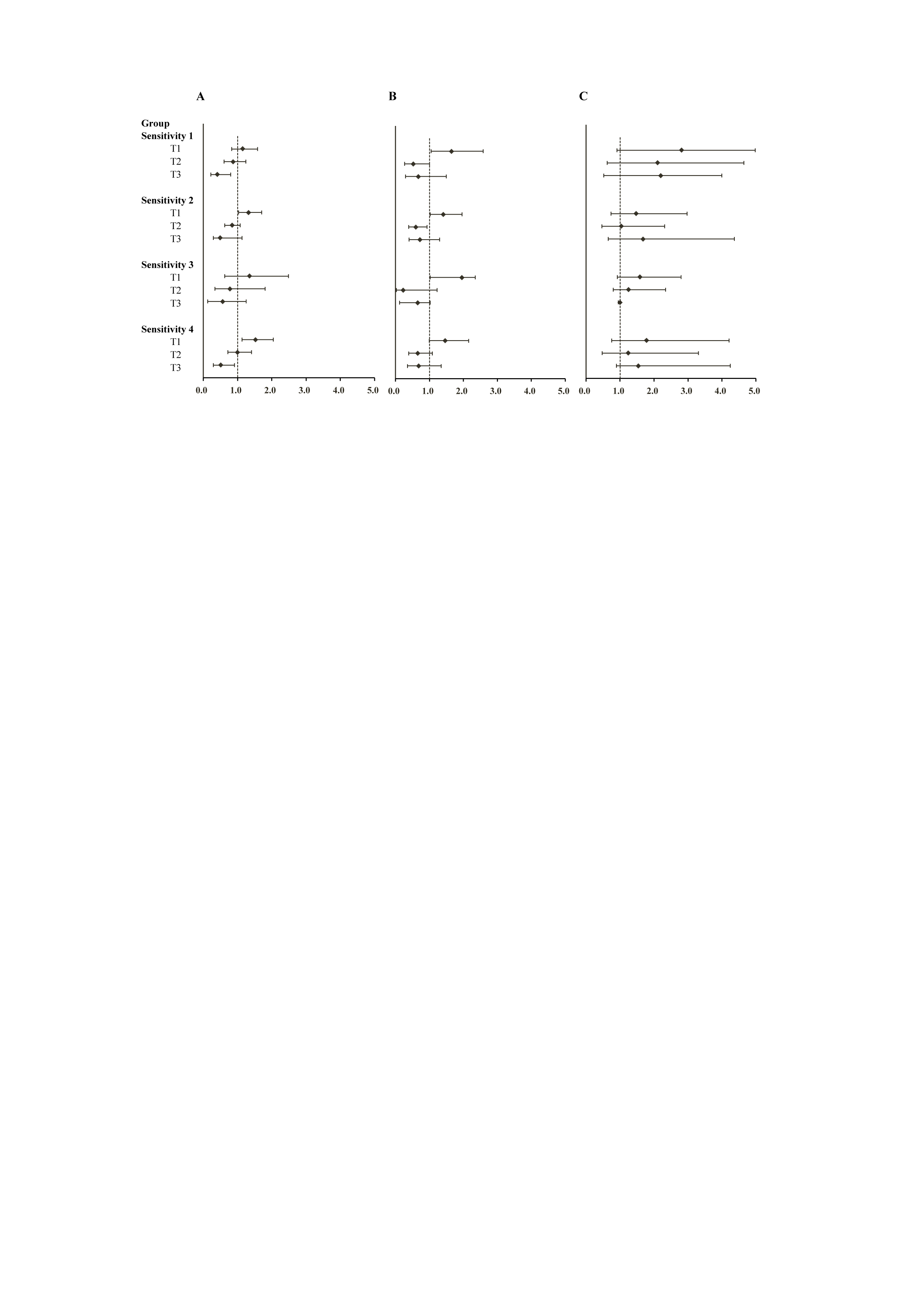

Supplement: Supplemental Material [file IANN_A_2427907_SM2499.zip › suppl_data/Figure S2.tif]

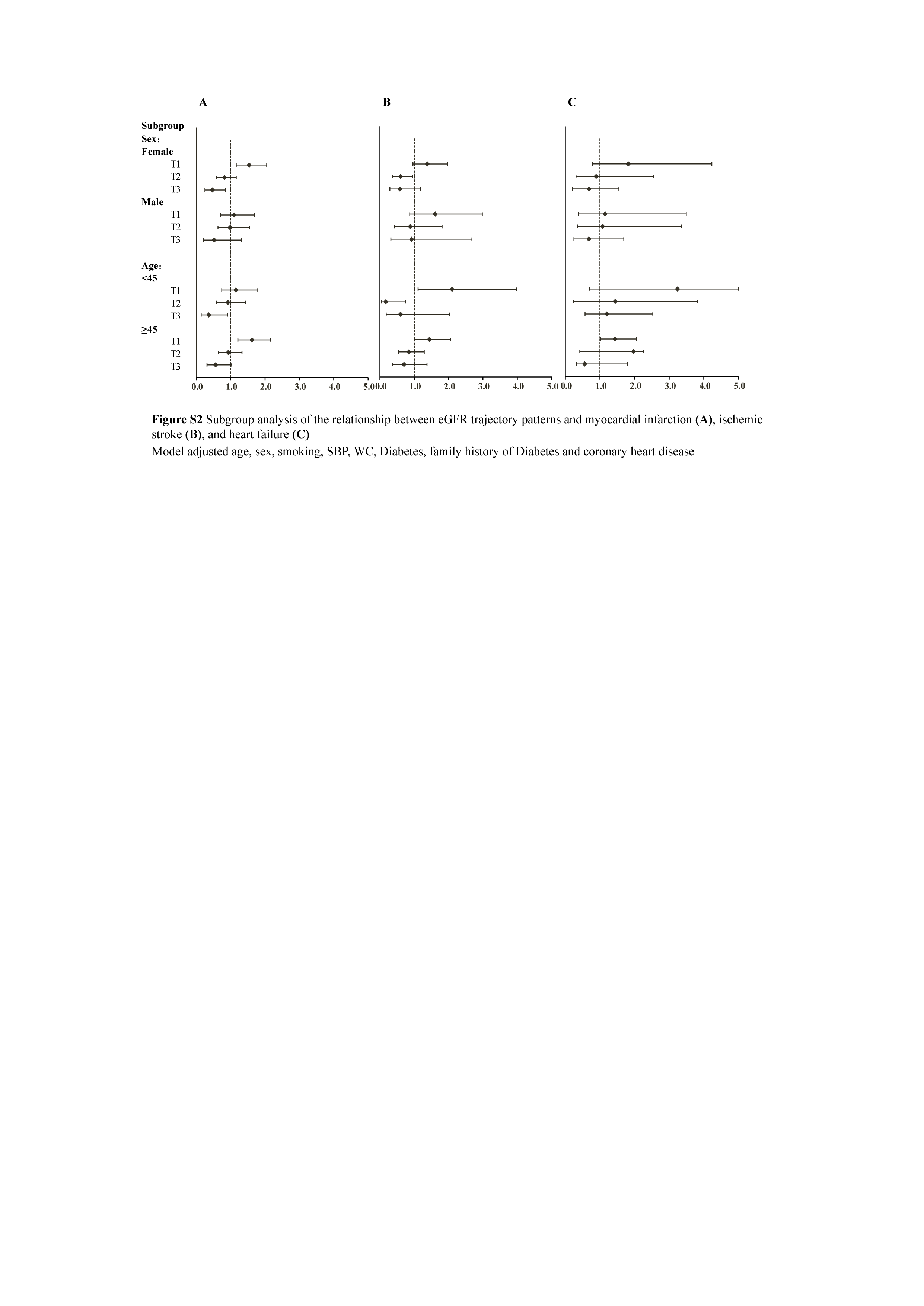

Supplement: Supplemental Material [file IANN_A_2427907_SM2499.zip › suppl_data/Figure S3.tif]
